# Chain Structure in a Cross-Linked Polyurethane Magnetic Elastomer Under a Magnetic Field

**DOI:** 10.3390/ijms20122879

**Published:** 2019-06-13

**Authors:** Mayuko Watanabe, Yoshihiro Takeda, Takayuki Maruyama, Junko Ikeda, Mika Kawai, Tetsu Mitsumata

**Affiliations:** 1 Graduate School of Science and Technology, Niigata University, Niigata 950-2181, Japan; w-myk.265@i.softbank.jp (M.W.); j-ikeda@sanyo-trading.co.jp (J.I.); mikagoro@eng.niigata-u.ac.jp (M.K.); 2 ALCA, Japan Science and Technology Agency, Tokyo 102-0076, Japan; 3 Rigaku Corporation, Tokyo 196-8666, Japan; y-takeda@rigaku.co.jp; 4 Bridgestone Corporation, Tokyo 187-8531, Japan; takayuki.maruyama@bridgestone.com; 5 Nihon Rufuto Corporation, Tokyo 110-0015, Japan

**Keywords:** stimuli-responsive material, magnetic elastomer, magnetic gel, computed tomography, viscoelastic property, magnetorheology

## Abstract

The morphology of magnetic particles with a size of 7.0 μm was observed for magnetic elastomers with a concentration of magnetic particles of 70 wt% using an X-ray microscope remolded into high resolution. Computed tomography images revealed that magnetic particles were distributed isotopically in the absence of a magnetic field, but they formed a chain structure in the polyurethane network under a magnetic field of 270 mT. It was also established, by image analysis, that magnetic elastomers had an anisotropic structure under the magnetic field.

## 1. Introduction

Magnetic elastomers are soft materials that are responsive to magnetic fields and consist of polymeric matrixes and magnetic particles. When magnetic fields are applied to a magnetic elastomer the viscoelastic properties alter in response to the magnetic field [1,2,3,4]. This is called the magnetorheological effect. We developed a new class of magnetic soft materials that exhibited drastic and reversible changes in the dynamic modulus by weak magnetic fields [5,6,7]. In addition to the viscoelastic property, the magnetic field response of physical properties such as surface property [8,9], electric conductivity [10,11], thermal conductivity [12,13,14], have also been reported. These changes in physical properties by magnetic fields are confirmed results of the change of the inner structure of magnetic elastomers.

It is widely accepted for magnetic fluids that the increase in the viscosity by magnetic fields is due to the chain formation of magnetic particles which align in the magnetic field. On the other hand, it is not easy to observe the chain structure of magnetic particles in cross-linked polymers such as gels and elastomers. There are several methods to observe the structure of magnetic particles, e.g., optical microscope, scanning electron microscope (SEM), or computed tomography (CT) observation. In magnetic fluids the magnetic particles move freely in the fluid and, therefore, a clear chain structure can be observed using an optical microscope with transmitted light. However, microscope observation is not useful for seeing the chain structure because light does not transmit through samples of magnetic elastomer with high concentrations of magnetic particles, where continuous chains are formed. In general SEM observation it is difficult to insert a permanent magnet in the microscope, therefore one cannot take photos of magnetic elastomers under magnetic fields. However, recently Lee et al. reported clear SEM photographs in which a spike structure appears by magnetic field on the surface of the magnetic elastomer [8]. This result strongly indicated that magnetic particles align in the direction of magnetic fields even if the matrix is a cross-linked polymer. 

Many researchers have tried to visualize the chain structure of magnetic particles under magnetic fields. At the beginning of this study, magnetic elastomers cured under a magnetic field were usually employed as a sample for SEM observation. However, the structure was formed before the cross-linking, and the resultant structure was different from the on-file structure of magnetic elastomers after cross-linking. In 2007 Chen et al. synthesized magnetic elastomers under various magnetic fields and carried out SEM observations and rheological measurements [15]. They reported a finite-column model that related microstructure and viscoelasticity, and revealed that the experimental data could be explained by the model. In 2012 Borin et al. measured the storage modulus on the cure process for magnetic fluids under a magnetic field of 220 kA/m [16]. They reported that the concentration of magnetic particles (<45 wt%) affected the particle morphology after cross-linking. In 2012 Gunther et al. observed by X-ray μ-CT the inner structure of magnetic elastomers cured under a magnetic field [17]. Borbath et al., also in 2012, observed by X-ray μ-CT the microstructure of magnetic elastomers cured under various magnetic fields of <220 kA/m and obtained quantitative information such as the size distribution of chains [18]. In 2013 Gundermann et al. observed by X-ray μ-CT the microstructure of magnetic elastomers with ~10 wt% magnetic particles cured under a magnetic field of 250 mT [19]. They made a comparison between the macroscopic change and the displacement of individual particles. Besides these studies, in situ observation has also been carried out. In 2007 and 2008 Stepanov et al. used an optical microscope with transmitted light to reveal that magnetic particles can move and form a chain structure when a magnetic field of 0.02 T is applied [20,21]. It was also shown that magnetic particles returned to their original position after the removal of the magnetic field. In 2014 Gundermann et al. observed magnetic elastomers containing magnetic particles (2 wt%) with a diameter of 35 μm using an X-ray μ-CT [22] under the following conditions: resolution 1 pixel = 3.2 μm, acceleration voltage 90 kV, current 170 μA, exposure time 4 s and 6.5 s. They showed that the combination of CT and digital image processing provided a tool for a quantitative analysis of single particle motion in a magnetorheological elastomer. They also observed the microstructure of magnetic elastomers with various concentrations of magnetic particles by X-ray μ-CT and, in 2017, tried to find the relation between macroscopic elasticity and the microscopic structure of magnetic particles [23]. Observations of the microstructure of magnetic elastomers using CT are in progress. In 2018 Sánchez et al. observed particle morphology at various magnetic fields on the magnetization curve of magnetic elastomers containing particles of neodymium magnet [24]. Pessot et al., also in 2018, succeeded in estimating the number of chains and the chain length, from CT images, and estimated the dynamic modulus and hardening effects [25]. However, the chain structure and the process of chain formation are still not clear for magnetic elastomers with high concentrations of magnetic particles. CT is a powerful tool for analyzing the inner structure of magnetic elastomers, as seen in the results of Odenbach’s group. In general, it is difficult to take clear photos of magnetic elastomers with high concentrations of magnetic particles due to the significant attenuation of the X-ray.

In this work, we tried to take photographs of the chain structure of magnetic elastomers with high concentrations of magnetic particles using remodeled high-resolution CT. The relation between the chain structure of magnetic particles and the magnetorheological effect is discussed.

## 2. Results and Discussion

**Figure 1:**
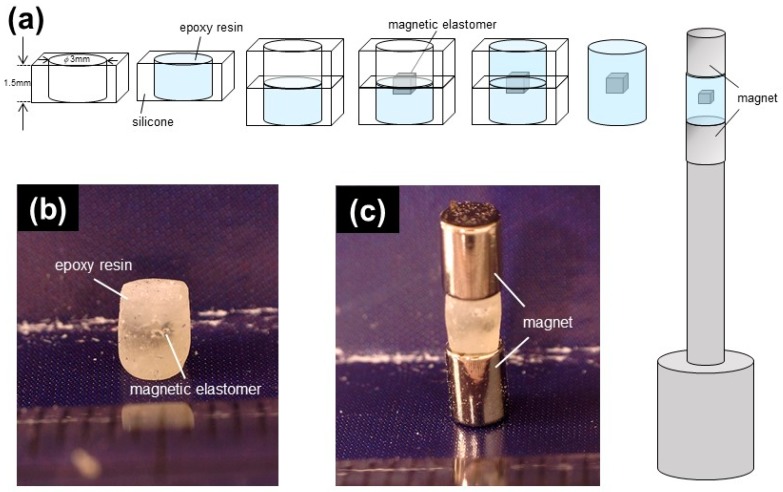
(**a**) Schemes of sample preparation for CT observations; a piece of magnetic elastomer was placed in epoxy resin. Photos of samples at (**b**) 0 mT and (**c**) 270 mT; sample was sandwiched by two permanent magnets with a magnetic field of 490 mT.

Figure 1a shows the schemes for preparing samples for CT observation. First, epoxy resin was placed in a silicone mold with a size of ϕ3 mm and a height of 1.5 mm. The other silicone mold was put on and a piece of magnetic elastomer was added to the surface of the epoxy resin. Epoxy resin was placed again in the upper mold. The epoxy was perfectly solidified after 60 min. Finally, the silicone mold was removed from the epoxy resin. Figure 1b exhibits the sample used in CT observation at 0 mT. Magnetic elastomer was placed in an epoxy resin. Figure 1c shows the sample used in CT observation under a magnetic field. The epoxy resin (compression modulus ~0.1 GPa) was sandwiched by two permanent magnets with a magnetic field of 490 mT, as shown in the figure. A pair of permanent magnets was used at the top and bottom of the epoxy resin, therefore, the geometry was similar to an electromagnet with two poles. The gap was 3 mm, and the sample size was 1 mm^3^. We assumed that the magnetic field near the magnetic elastomer was uniform. In reality, the orientation direction was parallel to the magnetic field, as shown later. We made a plastic assembly with a small hole for a tesla meter to measure the magnetic field strength. We measured the magnetic field strength in the hole in the exact same place as the magnetic elastomer within the epoxy resin; the magnetic field strength was 270 mT.

**Figure 2:**
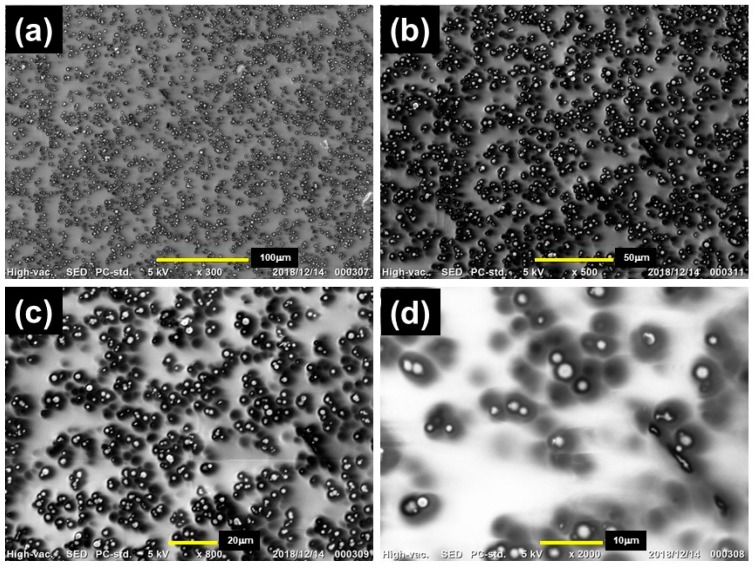
SEM photographs of a cross-section of the magnetic elastomer used in the CT observations at various magnifications; (**a**) ×300, (**b**) ×500, (**c**) ×800, (**d**) ×2000 (CI 70 wt%, DOP 70 wt%).

Figure 2 displays the SEM photographs of a cross-section of the magnetic elastomers used in the CT observations at various magnifications. Magnetic particles were randomly macroscopically dispersed in the polyurethane matrix and there were fewer agglomerates of magnetic particles. As we reported previously [26], clusters consisting of few magnetic particles, as seen in Figure 2d, remained even though sonication was carried out. The rheological data of this elastomer showed two features showing the random dispersion of magnetic particles. One was the storage modulus at the linear viscoelastic regime. The storage modulus at the linear viscoelastic regime (γ = 10^−4^) for magnetic elastomers was 1.9 × 10^4^ Pa, which is very close to that calculated via the Guth–Gold formula (= 1.3 × 10^4^ Pa) [27]. This strongly suggested a lack of apparent agglomerates, such as a particle network, in the magnetic elastomer. The other was the nonlinear viscoelasticity. We analyzed the particle dispersibility from a parameter showing nonlinear viscoelasticity β, which was obtained by the strain dependence of the storage modulus [7,26,28]. The nonlinear parameter β was defined as the following equation [6,8]:

(1) $$\beta \equiv 1-\frac{G^{\prime }\left(\gamma =1\right)}{G^{\prime }\left(\gamma =10^{-4}\right)},$$

where G’(γ = 1) and G’(γ = 10^−4^) are the storage modulus at strains of 1 and 10^−4^, respectively. The value of β for magnetic elastomers used in CT observation was 0.36. For example, the β for magnetic elastomers containing barium ferrite particles was 0.9; the β for magnetic elastomers containing carbonyl iron particles without sonication was 0.56. Furthermore, magnetic elastomers with high values of β demonstrated clear aggregations of magnetic particles. Accordingly, magnetic particles in magnetic elastomers used in the CT observation randomly dispersed in the polyurethane matrix at the microscopic scale [28].

**Figure 3:**
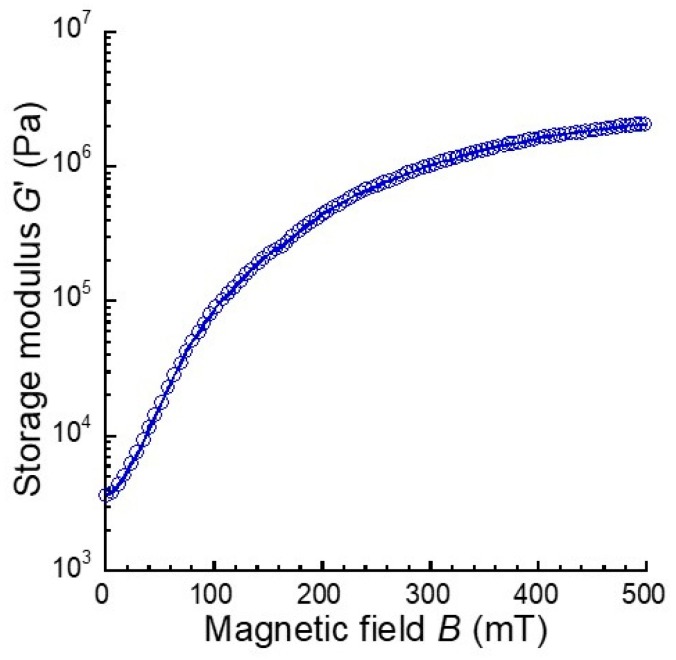
Magnetic field dependence of the storage modulus for magnetic elastomers used in CT observations (CI 70 wt%, DOP 70 wt%).

Figure 3 shows the magnetic field dependence of the storage modulus of the magnetic elastomers used in the CT observations. At 270 mT, which is equal to the magnetic field strength of the CT observation, the storage modulus of the magnetic elastomers was 8.2 × 10^5^ Pa and it corresponded to 40% of the maximum storage modulus (= 2.1 × 10^6^ Pa), where the magnetic particles fully aligned in the magnetic field direction. This value relating to the degree of alignment was too small for the observation of the alignment of magnetic particles. However, we could not apply a strong magnetic field in the CT experiment since the sample space around the sample stage was very limited. The storage modulus for the polyurethane elastomer without magnetic particles was 4.6 × 10^3^ Pa.

**Figure 4:**
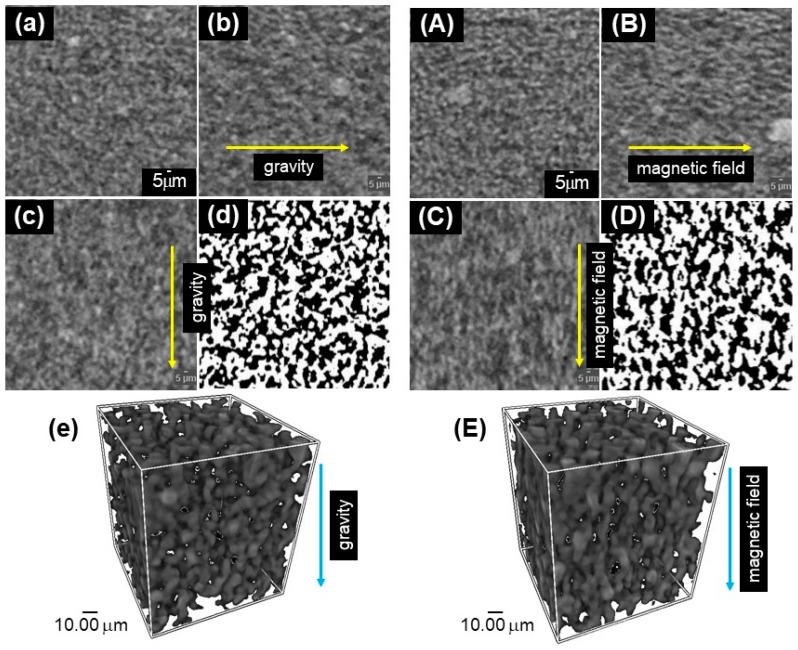
CT images for magnetic elastomers at (**a**–**e**) 0 mT and (**A**–**E**) 270 mT with different views; (**a**,**A**) top, (**b**,**B**) side, (**c**,**C**) front, and (**e, E**) 3D view. The directions of gravity and magnetic field are indicated by arrows. (**d**,**D**) Black-and-white images of the side view (CI 70 wt%, DOP 70 wt%).

Figure 4a–c,A–C demonstrate the CT images with different views for magnetic elastomers at 0 and 270 mT, respectively. Figure 4e,E show the 3D images of CT images for magnetic elastomers at 0 and 270 mT, respectively. The directions of gravity and the magnetic field are indicated by arrows in the figures. Note that the direction of gravity is the direction of the CT observation, which is not the direction of gravity at synthesis. Figure 4a,A demonstrate the view observed from the top of the magnetic elastomer. No apparent change in the morphology of the magnetic particles by the magnetic field can be seen in these photographs. Alternatively, both the side views (Figure 4b,B) and front views (Figure 4c,C) demonstrate a clear magnetic field effect on the particle morphology; that is, a chain structure was observed at 270 mT, as reported by Stepanov et al. [20,21]. They revealed using an optical microscope with transmitted light that magnetic particles can move and form a chain structure by applying a magnetic field of 0.02 T. As far as we know, our photos are the first to observe chain structures with high concentrations of magnetic particles. Magnetic particles are able to make a chain structure even at high concentrations of magnetic particle higher than 70 wt%. Similar structures, in which magnetic particles aligned in the direction of the magnetic field, were observed for some magnetic elastomers obtained from different syntheses. We also observed magnetic elastomers with carbonyl iron particles with 2.5 μm, however, the image was not clear. Transversely, it was easy to observe the morphology of magnetic particles larger than 100 μm by conventional CT. However, it was difficult to find chains consisting of only 10 magnetic particles within a 1 mm^3^ magnetic elastomer (the size should be smaller than 1 mm^3^ so that X-ray can transmit through the sample). Figure 4c shows how the magnetic particles seem to be linked in the direction of gravity. It was confirmed that the precipitation of magnetic particles at synthesis did not affect the textured appearance because the cross-linking reaction occurred within a few minutes. The concentration gradation of the magnetic particles was not observed in the macroscopic view of the CT image. We proposed two possibilities for the texture. One possibility was that the magnetic particles made an aligned structure induced by the deformation of the magnetic elastomer due to the solidification of the epoxy resin during sample preparation. However, the apparent deformation or elongation of the magnetic elastomer was not observed in the CT image. The other possibility was that it was due to the terrestrial magnetism, although the strength of the magnetic field was approximately 0.046 mT, which is too small to move magnetic particles within high viscose medium. Figure 4d,D indicate the black-and-white images of Figure 4c,C, respectively. Figure 4D at 270 mT clearly shows the chain structure of magnetic particles. However, no anisotropic structure was observed in Figure 4d at 0 mT. The chain-like structure seen in Figure 4c is discussed again later.

**Figure 5:**
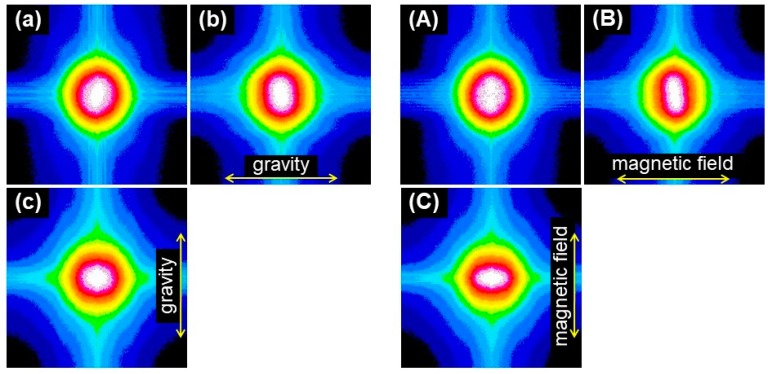
FFT images of CT photographs for magnetic elastomers with different views, (**a**,**A**) top, (**b**,**B**) side, and (**c**,**C**) front view. The direction of gravity and the magnetic field are indicated by arrows. (CI 70 wt%, DOP 70 wt%).

Figure 5 displays the fast Fourier transformed (FFT) images of the CT photographs of the magnetic elastomers presented in Figure 4. The direction of gravity and magnetic field are shown by arrows in the figure. Similar to Figure 4, Figure 5a,A are the top view, Figure 5b,B are the side view, Figure 5c,C are the front view. At 0 mT, concentric circles can be seen in the FFT images. However, the ring in Figure 5b seemed to collapse slightly. This was caused by the orientation of the magnetic particles along with the direction of gravity discussed in Figure 4. It is quite natural because the image was obtained from Figure 4; however, the asymmetric ring was observed in the FFT images parallel to the magnetic field. We considered the asymmetry to be due to the chain formation of the magnetic particles. We assumed that the contact between the magnetic particles was formed in the direction perpendicular to the magnetic field. However, clear anisotropy was not observed in Figure 5A.

**Figure 6:**
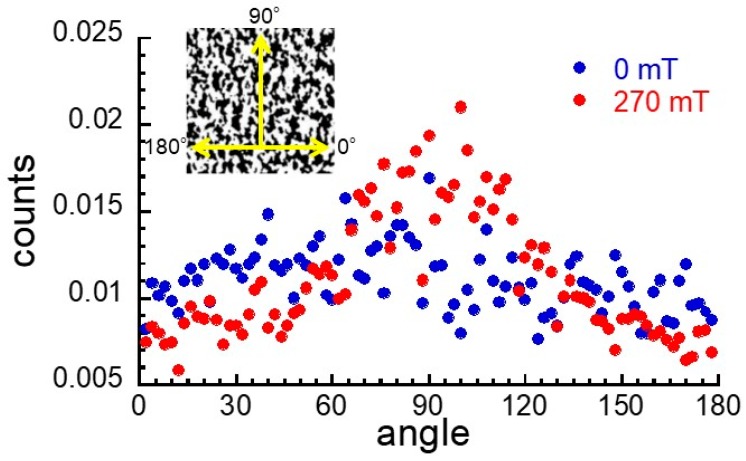
Angle distribution for magnetic elastomers at 0 (left) and 270 mT (right) (CI 70 wt%, DOP 70 wt%).

Figure 6 exhibits the distribution of angle for the boundary between the magnetic particle and the matrix of the magnetic elastomers at 0 and 270 mT. The angle was determined by the black-and-white images of Figure 4d,D using ImageJ software. The frequency was independent of the angle at 0 mT while it demonstrated a broad peak at around 90°. This strongly indicated that magnetic particles had isotropic distribution in the absence of a magnetic field and aligned in the direction of the magnetic field in the presence of a magnetic field. Gundermann et al. carried out the CT observations of the magnetic elastomers containing 2 wt% magnetic particles with a diameter of 35 µm and reported that magnetic particles were able to move within the cross-linked matrix. We could not estimate the moving distance of the magnetic particles in this experiment, although Odenbach et al. tried. However, in this experiment, we revealed that magnetic particles were able to move and form a chain structure even in a cross-linked matrix at high concentrations of magnetic particles. Experiments concerning magnetic field dependence or volume fraction dependence of the chain length are in progress but we feel the need to improve the resolution of CT to observe magnetic particles with micron sizes.

## 3. Materials and Methods

### 3.1. Synthesis of Magnetic Elastomer

Polyurethane elastomers and magnetic elastomers were synthesized by a prepolymer method. Polypropylene glycols (*M*w = 2000, 3000), prepolymers cross-linked by tolyrene diisocyanate (Wako Pure Chemical Industries. Ltd., Osaka, Japan), dioctyl phthalate (DOP, Wako Pure Chemical Industries. Ltd., Osaka, Japan), and carbonyl iron (CS Grade BASF SE., Ludwigshafen am Rhein, Germany) particles were mixed in a mechanical mixer for several minutes. The molar ratio of –NCO to –OH group for the prepolymer was constant at 2.01 (=[NCO]/[OH]). The median diameter of carbonyl iron particles was 7.0 ± 0.2 μm, determined by a particle size analyzer (SALD-2200, Shimadzu Co. Ltd., Kyoto, Japan). The saturation magnetization of carbonyl iron particles was measured to be 190 emu/g by SQUID magnetometer (MPMS, Quantum Design Inc., San Diego, CA, US), and the magnetization at 270 mT was determined to be 151 emu/g from the magnetization curve. Sonication was carried out for 5 min using an ultrasonic homogenizer (UD-211, Tomy Seiko Co. Ltd. Tokyo, Japan). The frequency was 20 kHz and the output power was 100 W. The mixed liquid was poured into a silicon mold and cured on a hot plate for 20 min at 100 °C. The weight concentration of DOP to the matrix without magnetic particles was fixed at 70 wt%. The weight fraction of the magnetic particles was kept at 70 wt%, which corresponded to a volume fraction of 0.23. The volume fraction of magnetic particles was calculated from the weight in feed and the density of constituents using the densities of magnetic particles (=7.565 g/cm^3^) and polyurethane (=1.0 g/cm^3^). On the other hand, the measured density for the magnetic elastomer was 2.555 g/cm^3^, accordingly the volume fraction was determined to be 0.24, which coincided with the volume fraction calculated from the weight in the feed.

### 3.2. Rheological Measurements

Magnetic field dependence of the storage modulus of the magnetic elastomers was measured by dynamic viscoelastic measurements using a rheometer (MCR301, Anton Paar Pty. Ltd., Graz, Austria) with a nonmagnetic parallel plate (PP20/MRD). The measurement was carried out at 20 °C. The strain was constant at 10^−4^, and the frequency was constant at 1 Hz. The sample was a disk 20 mm in diameter and 1.5 mm thick.

### 3.3. SEM Observations

Scanning electron microscope (SEM) observations were carried out using a JCM-6000 Neoscope (JEOL Ltd. Tokyo, Japan) with an accelerating voltage of 5 kV without Au coating. Magnetic elastomers with a volume fraction of 0.23 were used.

### 3.4. CT Observations

Computed tomography (CT) scanning observations were carried out using an X-ray microscope (nano3DX, Rigaku Co. Tokyo, Japan) remolded to high resolution. The CT images were generated with a 0.225° angular increment for a tube current of 20 mA and an acceleration voltage of 60 kV with a tungsten target. The exposure time was 16 s and the resolution was 1.0 micron/pixel. The sample of the magnetic elastomer was cut into a cube with the dimensions 1.0 mm × 1.0 mm × 1.0 mm, and was embedded in an epoxy resin (3.0 mm in diameter, 3.0 mm in height) as shown in Figure 1. The strength of the magnetic field around the magnetic elastomer was 270 mT by a tesla meter (TM-601, KANETEC Co., Ltd. Nagano, Japan). 

### 3.5. D-FFT Analysis

To visualize the alignment of the magnetic particles clearly, 2D-fast Fourier transformation (FFT) analysis was performed on the CT images taken at 0 and 270 mT using ImageJ software [29]. The obtained FFT images were on a 2D-power spectrum that masked low frequencies.

## 4. Conclusions

The morphology of magnetic particles with a size of 7.0 μm was observed for magnetic elastomers with a concentration of magnetic particles of 70 wt% using an X-ray microscope remodeled from conventional CT into high resolution. The computed tomography images strongly indicated that magnetic particles had isotropic distributions in the absence of magnetic fields and that the magnetic particles aligned in the direction of the magnetic field in the presence of a magnetic field. We also carried out image analysis and found that magnetic elastomers had an anisotropic structure under the magnetic field. We believe that detailed analysis for the chain structure using computed tomography images is a breakthrough for designing the structure of a polymer network for magnetic elastomers in the next generation.
